# Supplementary material for: The operational environment and rotational acceleration of asteroid (101955) Bennu from OSIRIS-REx observations
Source: Nat Commun. 2019 Mar 19;10:1291. doi: 10.1038/s41467-019-09213-x (PMC6425024; doi:10.1038/s41467-019-09213-x)
Supplement: Supplementary file 1 — Supplementary Information [file 41467_2019_9213_MOESM1_ESM.docx]

| Date and Time (UTC) | Instrument | Start and End Range of Search (km) | Phase Angle  (degrees) | Distance from Bennu Searched (km) | Smallest Detectable Diameter (cm) | Smallest Detectable Diameter (cm) | Smallest Detectable Diameter (cm) |
| --- | --- | --- | --- | --- | --- | --- | --- |
|  |  |  |  |  | Manually Blinking | Co-added Images | Catalina Sky Survey moving object detection software |
| 23-Oct-2018 04:00-09:00 | PolyCam | 3131-3036 | 36 | 60 | 49 | N/A | 42 |
| 24-Oct-2018 04:00-09:00 | PolyCam | 2679-2585 | 36 | 51 | 42 | N/A | 37 |
| 25-Oct-2018 04:00-09:00 | PolyCam | 2228-2134 | 37 | 42 | 35 | N/A | 30 |
| 26-Oct-2018 04:00-09:00 | PolyCam | 1778-1684 | 37 | 33 | 28 | 20 | 24 |
| 27-Oct-2018 04:00-09:00 | PolyCam | 1328-1234 | 36 | 24 | 21 | 15 | 18 |
| 28-Oct-2018 04:00-09:00 | PolyCam | 880-787 | 34 | 15 | 13 | 10 | 11 |
| 30-Oct-2018 04:00-09:00 | MapCam | 216-215 | 7 | 20 | 8 | N/A | N/A |
| 31-Oct-2018 04:00-09:00 | MapCam | 210-209 | 6 | 20 | 8 | N/A | N/A |
| 10-Nov-2018 04:00-09:00 | PolyCam | 156-155 | 11 | 4 | 10 | N/A | N/A |
|  | MapCam | 156-155 | 11 | 19 | 8 | N/A | N/A |
| 11-Nov-2018 04:00-09:00 | PolyCam | 153-153 | 15 | 4 | 10 | N/A | N/A |
|  | MapCam | 153-153 | 15 | 19 | 8 | N/A | N/A |

Supplementary Table 1 – **Satellite search details.** Dates, instruments, observing circumstances and size limits of satellites detectable by different search methods. Observations were obtained between 04:00 and 9:00 UTC on each date. The phase angle, distance from Bennu searched, and smallest detectable diameters are for the end of the daily observations at 9:00 UTC.

| **Lommel-Seeliger Model** | | | | | | | |
| --- | --- | --- | --- | --- | --- | --- | --- |
| **Filter** | **Wavelength** | ***P*** | ***p*_1_ (10^-2^)** | ***p*_2_ (10^-4^)** | ***p*_3_ (10^-6^)** | **Bond albedo** |  |
| V | 549.5 | 0.0443 | -3.77 | 3.34 | -2.35 | 0.0170 |  |
| **IAU HG Model** | | | | | | | |
| **Filter** | **Wavelength** | ***H*** | ***H* Error** | ***G*** | ***G* Error** |  |  |
| V | 549.5 | 20.209 | 0.027 | -0.031 | 0.009 |  |  |
| **H, G1, G2 Model** | | | | | | | |
| **Filter** | **Wavelength** | ***H*** | ***H* Error** | ***G*_1_** | ***G*_1_ Error** | ***G*_2_** | ***G*_2_ Error** |
| V | 549.5 | 20.777 | 0.124 | 1.602 | 0.249 | -0.214 | 0.055 |
| **H, G12 Model** | | | | | | | |
| **Filters** | **Wavelength** | ***H*** | ***H* Error** | ***G*_12_** | ***G*_12_ Error** |  |  |
| V | 549.5 | 20.408 | 0.016 | 1.162 | 0.031 |  |  |

Supplementary Table 2 – **Phase Function Photometry Model Parameters.** Fit parameters for various methods of modeling the shape of Bennu’s phase function (magnitude vs. phase angle). Wavelengths are in nanometers. All other parameters are unitless.
